# Prophylaxis of Lobar Pneumonia

**Published:** 1905-07-22

**Authors:** 


### Hospital Clinics.

### PROPHYLAXIS OF LOBAR PNEUMONIA.

"Pneumonia being the most dreaded of all human diseases owing to its unparalleled death-rate and universal prevalence, obviously its prevention becomes a subject of first importance." With these words Dr. J. M. Anders [1] opens a plea for more serious attempts to limit the ravages of this disease. The object is a worthy one, though we suspect that the general dread of the disease does not reach the heights indicated by the author. A malady which, though among the most fatal of human ailments, yet comparatively seldom leaves us scotched and crippled, as for instance, does rheumatic fever, gains a sort of sympathy, from the initiated at all events, even though by custom they still pray for deliverance from sudden death. Supposing that a dispensation of providence gave Dr. Anders a freedom of choice between pneumonia and, let us say, locomotor ataxy, or melancholia, or leprosy, as an instrument for his own chastening, we can hardly believe that he would remain of his present opinion.

This exception is taken in no mere carping spirit; the amount of labour lavished on the prevention of any disease stands in direct proportion to the popular dread of it, and fear thus becomes the basis of the whole matter. Now pneumonia being a malady due to a well-known organism, it is natural that on *a priori* grounds it should be held preventible, but a little reflection accentuates the grave difficulties encountered by practical attempts at prophylaxis. The preventibility of any microbic disease is proportional to the readiness with which its causative organism can assume a saprophytic existence. Thus it is clear that an organism like the gonococcus, which is an obligatory parasite, as is that of syphilis, can only be transmitted from person to person by actual contact. Gonorrhœa and syphilis therefore, are the most absolutely preventible of all diseases, as they are, unhappily, the least prevented. But, when an organism can readily assume a saprophytic phase, as does the pneumococcus, its sphere of potential action is immensely increased, especially when it is that of an infection primarily air-borne. Such an organism, discharged by the expiratory effort of the sick man, may exist for some time in air or dust: its actual virulence is diminished but its potentialities remain unimpaired: presently, being inhaled by another, it becomes lodged in his air-passages, where its further action is determined by its own virulence and the susceptibility of its host. The danger is increased by the fact that the pneumococcus may exist and multiply on the mucous membranes of the insusceptible, and by them be ejected to produce its typical pathogenic effects upon some third subject of greater susceptibility, as is the case, in a lesser degree, with diphtheria. It is certain that the pneumococcus is ubiquitous.

On the other hand experience has established the fact that the spread of pneumonia in institutions for the sick is a rare contingency. House epidemics have been put on record, but they are undeniably rare in proportion to the frequency of pnemonia; and it must be admitted that the contagious nature of the disease is, in practice, but seldom demonstrable. The *a priori* argument therefore fails, for pneumonia is a disease whose contagion is generally indirect. The bearing of this fact upon prophylaxis diverts attention from the possible distributors of the affection and focusses it upon the possible recipients. The problem before us is to reduce the susceptibility of the healthy.

The practice of a children's hospital in a crowded quarter is an excellent field for the study of the infections depending upon the pneumococcus. In slum-bred children this organism produces a variety of lesions which are seldom seen elsewhere, in addition to the usual ones here seen in great abundance. The pneumococcus shares indeed with the tubercle bacillus the responsibility for a very large majority of the total of deaths in childhood, and for infinitely more lesions of less gravity. Besides lobar pneumonia, the pneumococcus can claim credit for the majority of bronchopneumonias secondary to measles and whooping-cough, for most empyemas, for most middle-ear suppurations, for most purulent infections of joints, for many purulent rhinorrhœas, and for many purulent invasions of the meninges, pericardium and peritoneum. Now it is a curious thing that the only one of these lesions frequently met with among children of the better classes is lobar pneumonia. To take the case of pneumococcal empyema alone, at the hospital in question this disease leads to not less than one operation a week, yet we have it on the authority of a children's specialist enjoying a large practice for many years, that he could count on his ten fingers the number of empyemas he has met with in private practice among children of better circumstances. The only possible deduction from these facts is that the difference depends upon general hygiene. Pneumococcal infections, as is pointed out by Dr. Anders, reach their maximum in the cold months of the year, at a time, that is to say, when, among the poor particularly, the need of warmth becomes a paramount consideration, and when the closure of all windows exaggerates the unwholesomeness of an already over-burdened air-space.

It is easy to frame ideal lines of prevention like those of Dr. Anders. For instance, one of his conclusions is to the following effect: "A large proportion of the general populace harbours the pneumococcus in the naso-pharynx, and this is especially true in families and institutions in which cases of pneumonia have occurred; hence thorough cleanliness and systematic disinfection of these chambers should be carried out during the pneumonia season." We think Dr. Anders must be sanguine of his fellow-men if he fancies they will submit to a daily "thorough disinfection" of their nasal passages for the avoidance of a disease whose contagion is so seldom demonstrable. It would moreover be interesting to learn how far such disinfection of these inaccessible passages is in practice possible.

In conclusion, it seems to us that the elimination of the pneumococcus is impossible, but that its ravages can be reduced to their lowest limits by thorough ventilation and good hygiene generally. The great obstacle to the prevention of infections is the abiding belief in the malignity of fresh air; this is the first superstition we of the profession must set ourselves to conquer: it is a creed not limited to the poor, though naturally the overcrowding chronic amongst the latter exaggerates the evils that attend the creed. If we can persuade people to live in well-ventilated rooms we shall do far more towards the suppression of pneumococcal infections than by any insistence upon the isolation of sufferers from pneumonia, or by such a rigid disinfection of their persons, bedding, and discharges as is recommended by Dr. Anders.

[1] Med. News, June 3, 1905.

